# Farce Comedy and Expert Evidence

**Published:** 1907-03

**Authors:** 


### Farce Comedy and Expert Evidence

THE trial of an apparently perverted individual with mental strabismus, if one may rely on the accuracy of the associated press reports, for the wanton killing of a man whom he believed to have been the cause of the moral ruin of a woman he later made his wife, has brought vividly to the public attention the dramatics and the farcical elements of expert evidence. The trial reeked with the drippings of the immorality of the artistic element of New York city; it stank with the bestiality of gem encrusted vice and then, after the legal foundation had been laid, came the experts.

The first to take the stand was Dr. C. E. Wiley. of Pittsburg, whose position was best expressed in his own plaintive plea during the grilling he received at the hands of District Attorney Jerome: "I came here as a witness of fact and they have made of me an expert without any preparation."

That poor wail of woe from the depths of a perturbed spirit is one of the strongest arguments the lay reader could wish in justification of his general denunciation of the average expert testimony. Dr. Wiley as a witness was an error; as an expert he was a gelatinous failure. Whether he was an expert or a witness of fact, he made statements which were beyond redemption; statements which if made by a sophomore in any medical school would have been sufficient to set him back for another year in the dissecting room. It is not worth while to go into details regarding the answers made by Dr. Wiley; it is sufficient to mention that when he was asked where the pneumogastric nerve entered the spinal cord, he replied, according to the published report, that it was in the lumbar region; he did not know the Romberg test; he could not tell its purpose or where it was mentioned; he confessed to an almost infantile ignorance of the textbooks covering the specialty he practised—mental and nervous diseases; he thought Argyl-Robertson were the names of two men who had done work on the eye; he did not know clearly what the Argyl-Robertson "eye test" was; he had never heard of the consensual test; he was absolutely in the dark regarding the anatomy of the nervous system; he could not tell what organs were supplied by the pneumogastric nerve nor tell its other name; nor could he give the origin or course of any of the cranial nerves. And he even permitted Mr. Jerome to quiz him regarding the function of the "lymphatic nerve" without correcting him. Whether Jerome was honest in speaking of the "lymphatic," having misunderstood the whispered question of one of his own experts, or whether he purposely mislead the witness is not known; but Dr. Wiley let slip a golden opportunity to play with the district attorney.

At the present writing, Dr. Britton D. Evans, superintendent of the insane asylum at Morris Plains, N. J., an expert for the defense, is occupying the center of the stage and undergoing cross examination at the hands of Mr. Jerome, who appears to be leading up to the point where he will compel the witness to declare that the prisoner is mentally incapacitated at the present time, upon which, presumably, to lay the foundation for an application for a commission in lunacy to determine the defendant's mental status.

Aside from the fact that Dr. Evans is patently on the stand for the purpose of proving that the prisoner was insane when he killed his man, and immediately became sane when his job as the self-elected agent of Providence was completed, he is a witness with whom the district attorney has had considerable trouble, for he knows his subject thoroughly and is a careful and not at all backward witness of the expert class. He answers when he thinks it advisable for the interests of his side and when he doesn't he just squirms, and in many instances has succeeded in escaping being pinned down to a "yes" or "no" answer. He has all the finesse of an expert and understands all the niceties of the legal loopholes through which such a witness, who keeps his wits about him, may wriggle when necessity arises. He has declared that the prisoner was suffering from a "brain storm" and a "mental explosion" at the time of the killing; but under the skilful guidance of the diplomatic Mr. Jerome he was led to the statement that the murderer had been mentally irresponsible prior to the killing and prior even to the time when he succeeded, after repeated effort, in obtaining the woman whom he afterward married. There are other experts to be heard and their evidence will be interesting reading.

As to the effect of Dr. Wiley's statements the worst they have done is to give rise to a new expression —a "wileyism"—indicative of a statement which on its face is either merely absurd or purely assinine. It has been explained in defence of Dr. Wiley's poor showing that he was suffering from stage fright. That may be so in a measure; but expert evidence in such a case would bring forth the fact that stage fright is characterised by silence—inability to speak; and not by the making of wholly hopeless answers of simple fact.

